# Brazilian adolescents’ knowledge and beliefs about abortion methods: a school-based internet inquiry

**DOI:** 10.1186/1472-6874-14-27

**Published:** 2014-02-13

**Authors:** Ellen MH Mitchell, Silke Heumann, Ana Araujo, Leila Adesse, Carolyn Tucker Halpern

**Affiliations:** 1 Amsterdam Institute for Global Health & Development, Amsterdam, Netherlands; 2 International Institute of Social Studies, Erasmus University Rotterdam, The Hague, The Netherlands; 3 Anthropology Department, Amherst College, Amherst, USA; 4 University of Rio de Janeiro School of Public Health, Rio de Janeiro, Brazil; 5 Carolina Population Center, University of North Carolina, Chapel Hill, USA

**Keywords:** Abortion, Adolescents, Brazil, Internet, Emergency contraception, Reproductive health, Pregnancy, Misoprostol

## Abstract

**Background:**

Internet surveys that draw from traditionally generated samples provide the unique conditions to engage adolescents in exploration of sensitive health topics.

**Methods:**

We examined awareness of unwanted pregnancy, abortion behaviour, methods, and attitudes toward specific legal indications for abortion via a school-based internet survey among 378 adolescents aged 12–21 years in three Rio de Janeiro public schools.

**Results:**

Forty-five percent knew peers who had undergone an abortion. Most students (66.0%) did not disclose abortion method knowledge. However, girls (aOR 4.2, 95% CI 2.4-7.2), those who had experienced their sexual debut (aOR1.76, 95% CI 1.1-3.0), and those attending a prestigious magnet school (aOR 2.7 95% CI 1.4-6.3) were more likely to report methods. Most abortion methods (79.3%) reported were ineffective, obsolete, and/or unsafe. Herbs (e.g. marijuana tea), over-the-counter medications, surgical procedures, foreign objects and blunt trauma were reported. Most techniques (85.2%) were perceived to be dangerous, including methods recommended by the World Health Organization. A majority (61.4%) supported Brazil’s existing law permitting abortion in the case of rape. There was no association between gender, age, sexual debut, parental education or socioeconomic status and attitudes toward legal abortion. However, students at the magnet school supported twice as many legal indications (2.7, SE.27) suggesting a likely role of peers and/or educators in shaping abortion views.

**Conclusions:**

Abortion knowledge and attitudes are not driven simply by age, religion or class, but rather a complex interplay that includes both social spaces and gender. Prevention of abortion morbidity and mortality among adolescents requires comprehensive sexuality and reproductive health education that includes factual distinctions between safe and unsafe abortion methods.

## Background

In Brazil abortion is routinely condemned in public discourse yet practiced privately on a large scale [1]. Adolescents, coming of age in such an atmosphere, are likely to hear conflicting information regarding the safety, efficacy, and acceptability of induced abortion methods. Adolescents’ perceptions are likely to be influenced by the moral evaluations of abortion articulated by peers, the media, family, educators, or by religious leaders [2,3]. Experts have recently highlighted the need to better understand adolescent’s information pathways regarding abortion methods as a means of reducing their risk-taking and protecting their health [4].

Legal abortion in Brazil is restricted to three situations: to save a woman’s life, in the case of rape, and for anencephalic pregnancies. Higher-income women avail safe abortion for other indications via private clinics while poorer women and adolescents tend to resort to less safe methods in less sanitary conditions [5,6]. Death due to unsafe abortion is the third leading cause of maternal mortality in Rio de Janeiro, concentrated in the poorest sectors of the city[7].

Brazilian adolescents are more vulnerable to coerced, unplanned and unprotected sex than adults [8,9]. An estimated 17-40% of first pregnancies end in abortion among urban young adults in Rio de Janeiro [10]. Studies in Ceará, Bahia, Pernambuco, Paraíba and Sao Paulo reveal that adolescents are disproportionately represented among those treated for complications of unsafe abortion. Although adolescents 15–19 years of age comprise only 16% of the population, adolescents make up over 22% of those who require emergency postabortion care [11-13]^a^. Adolescents are similarly over-represented among those needing legal abortion care as a result of rape or incest [8,14,15]. Although Brazil has many progressive health policies on paper, in practice Brazilian adolescents often face barriers to access to accurate reproductive health information and care, with limited recourse when their rights are violated [16,17].

A spate of abortion prevalence surveys have been published in the last three years, yielding divergent estimates of abortion among youth ranging from 0% to almost 60% of pregnancies [18-20]. While these studies have addressed some deficits in our understanding of the mechanics of abortion attempts, and crucially the role of social networks, most studies included only youth above the age of eighteen and often only females [8,19,21-23]. The data presented as “adolescent experiences” are reflections from a considerable distance, with the attendant recall bias. Moreover adolescent males’ reports of the prevalence of abortion often differ from that of female peers [24-26].

Historically, there has been considerable attention paid to the health outcomes of Brazilian adolescents admitted to public hospitals [27]. However, we know less about abortion attempts that do not result in hospitalization, giving a distorted picture of abortion methods and safety [21,27]. Queries of adolescents’ beliefs about the safety and efficacy of abortion procedures and behaviour using school or community-based samples are necessary to understand the actual diversity and heterogeneity of youth abortion attempts [21,27].

Over the past ten years, much research on abortion in Brazil has focused on self-medication with the prostaglandin analog misoprostol (“Cytotec™”) for both safe and unsafe abortions [19,27-30]^b^. Some experts have attributed the apparent decline in abortion-related mortality to the off-label use of misoprostol [31,32] Brazilian feminists, alarmed by the use of misoprostol, successfully lobbied for its restriction from sales in pharmacies in 1991. In 2006 the Brazilian government sought to further limit access to misoprostol by banning information about it on the internet [33]. Therefore, black market access to misoprostol can be prohibitively expensive for youth and often involve engaging with drug dealers and sellers of contraband [33]. At the time these data were collected, the cost of two pills on the black market averaged about US$35, half the monthly salary of many low-income Brazilians [28,29,34].

Small urban studies of adolescents’ abortion attempts show that many ineffective abortion attempts by adolescents involve herbal teas [26,35]. Herbal approaches to abortion are not novel, but may be culturally specific [36]. Despite Brazilians’ documented affinity for homeopathy and its official recognition by the government, “cocktail” abortion methods involving diverse homeopathic and over the counter (OTC) drugs have received little attention [37]. Some authors suggest that the use of such remedies was not conceptualized as abortive per se, but rather as menstrual regulation to bring on menses in the liminal period before a positive pregnancy diagnosis, a characteristic shared with misoprostol. In many contexts women frame these attempts as menstrual regulation rather than abortion to mitigate social stigma [33,38].

Abortion attitudes are dynamic and challenging to measure, as they are known to be heavily influenced by contextual factors and survey design effects [39-41]. Abortion is known to be a difficult topic to broach in both face-to-face interviews and self-administered paper surveys [42]. Both have yielded under-reporting and/or dubious conclusions [19,26,43,44]. The extent to which public discourse or political positions affect private behaviour is challenging to measure [45]. However research in Brazil and other contexts has often shown a striking discordance between expressed abortion attitudes and actual abortion behaviour [42,46]. Bailey and colleagues (2003) found that at least half of pregnant Brazilian adolescents surveyed had friends or family *recommend* abortion to manage an unwanted pregnancy. This private advice reflects a well-documented acceptance of abortion as an option in the case of a specific unwanted pregnancy, despite a public discourse of moral censure [26,46]. Bailey et al. (2003) have shown how Brazilian adolescents’ abortion attitudes can be quite dynamic and influenced by individual pregnancy outcomes.

Using a unique Internet methodology and a diverse sample of in-school adolescents in Rio de Janeiro, our study addressed gaps and limitations of prior inquiries by examining adolescent knowledge and perceptions of abortion methods outside of clinical settings. Previous research has suggested that computer-assisted research methods can be a more reliable means of collecting honest personal information on sensitive sexual health topics from Brazilian adolescents [44,47]. We used the lure and greater opportunity for candour of the internet to shed light on four research questions:

Are students aware of unwanted pregnancy and abortion behaviour in their peer group?

How much do adolescents know about abortion methods and their safety?

What do adolescents’ think the legal status of abortion for specific indications should be?

Which, if any, socio-demographic or school characteristics predict abortion knowledge and attitudes?

## Methods

This analysis was nested within an Internet-based longitudinal study of adolescents undertaken during 2003–2006 on a broader range of sexual and reproductive health topics [48]. The University of Campinas and the University of North Carolina at Chapel Hill School of Public Health Ethical Review Committees approved the study protocol. Three study schools in Rio de Janeiro were purposefully selected from a list of all public school on the basis of large size, equitable mix of race, gender, and social class, non-denominational curricula, and public funding. A university-affiliated magnet high school with a reputation for pedagogical innovation (*Colégio de Aplicação)*, and two state-funded technical schools (*Escola Técnica do Estado*), one upper secondary school and one lower secondary school, received computer hardware, internet access, staff training, filtering software, privacy cubicles, and key boarding classes [49]. All students and their parents received factsheets inviting them to participate and describing study objectives, procedures, risks, and web surfing benefits. Written parental consent was required for participants under 18 for the three modules on experiences of violence, sexual behaviour, and abortion.

Students completed six modules on-line (each module took on average of 6–8 weeks) over a 15 month period, which included holidays. Completion was concurrent and individually-paced. A new module was released after 80% of participants had completed the prior module. The final abortion module (#6) described here, queried students on their knowledge of abortion methods, perceived safety of different methods, and prevalence of pregnancy and abortion among peers, and knowledge of and opinions on Brazilian legal indications for abortion. Participants were also queried on personal pregnancy and abortion experiences, but these were infrequently reported. Students could opt-out of any question by responding that they did not know or chose not to answer.

After completing each questionnaire, students were given an additional 25 minutes to freely explore the Internet. Software filters precluded exposure to hate sites and pornography. Students were encouraged to visit sites with adolescent-appropriate health information on sexual and reproductive topics but few availed this option.

## Results

A total of 559 students (72.5%) enrolled in the study. Among adolescents who assented to join, 70.9% (n = 396) of parents gave consent for the ‘sensitive’ modules on sexuality, violence, and abortion. Out of these, 380 students completed the abortion module and comprise the sample. The socio-demographic characteristics of the three-school sample mirror the gender, race, age and sexual developmental profile of Rio’s public schools as a whole– however the socioeconomic status of the sample is higher [50] – see Table 1. Students were permitted to self-identify with as many racial heritages as they wished and 41.8% identified with two or more. Similarly, students could select more than one faith. In this sample, 97.0% of students had previously used a computer, 90.0% had used the internet and roughly half used the internet at least several times per week.

**Table 1:** Socio-demographic characteristics (n = 380)

	**Percentage of total respondents (%)**
Gender	
Girls	60.8
Boys	39.2
Age in years	
12-15	35.5
16-17	49.5
18-21	15.0
Race*	
White	66.6
Pardo (mixed heritage)	48.0
Black	21.9
Indigenous	16.6
Asian	3.7
Other	4.2
Socioeconomic status^‡^	
Low	19.6
Medium	51.2
High	29.2
Religious affiliation*	
Catholic	57.6
Evangelical protestant	25.1
Traditional protestant	11.1
African Brazilian	9.2
Other faiths	3.5
None	6.5
Religious practice	
Never	7.6
Less than once a month	35.5
Once a month or more, but less than once a week	11.8
Once a week or more	45.1
Maternal educational attainment	
Any primary	35.1
Any secondary school	39.7
Any University	25.2

*Race and religion proportions do not sum to 100% because students could chose more than one affiliation.

^‡^Socioeconomic status (SES) was assessed through the use of a proxy measure, an unweighted scale composed of a count of ownership of 18 household commodities [51]. The “low” SES category was defined as possession of thirteen commodities or fewer, “medium” as ownership of 14–16 items, and “high” as owning 17 or 18 commodities.

Almost half of students (45.0%) reported attending religious services on a weekly basis. More than a third (36.6%) reported having had their sexual debut.

Most students (69.5%) knew a peer who had experienced an unwanted pregnancy and 45.1% knew an age-peer who had had an abortion (Table 2). Superior maternal educational attainment and attendance at the magnet school were negatively associated with knowing age peers with both unwanted pregnancies and abortions. These results are similar to those documented by Garcia Castro et al. (2004: 220) who found that adolescents living in Brazilian major cities tended to know an age-peer who had had an abortion (ranging from 42.0% in Goiânia to 68.3% in Maceió) [52]. Girls were less likely to report sexual debut, but more likely to report knowing peers who had been pregnant and had aborted. Yet abortion method knowledge reports were less common (34.0%) among both genders, particularly among boys (18.9%).

**Table 2:** Peer pregnancy and abortion awareness, method reporting, and legal abortion attitude by gender, age, school, and sexual status

	***Do you know anyone your age who has had an unwanted pregnancy?***	***Do you know anyone your age who has had an abortion?***	***Have you heard or read about different methods to end an unwanted pregnancy?***	***When do you think termination of pregnancy should be legal *?**
				**Legal abortion attitude scale**
				**Mean (S.E.)**
				**Range 0-8‡**
**Total (% or mean)**	69.5	45.1	34.0	2.1(.10)
**Gender**				
Girls (214)	73.5*	53.9*	44.0*	2.2 (.13)
Boys (131)	**63.3**	**31.5**	**18.9**	2.0 (.18)
**Age**				
≤ 14 years (124)	52.8*	31.5	28.9	1.9 (.18)
15-16 years (168)	80.6	51.8	39.9 Φ	2.2 (.14)
≥17 years (54)	76.4	59.3	28.1	2.2 (.27)
**Maternal education**				
Any primary	37.3	39.9	32.3	2.0 (1.8)
Any secondary	44.3	42.0	39.5	2.2 (.17)
Any University	18.7*	18.2*	28.2	2.2 (.21)
**School**				
Technical middle school (62)	**65.1**	**39.7**	**17.6**	1.4 (.12)*
Technical high school (227)	**79.9**	**55.7**	**38.2**	**2.1 (.25)**
Magnet high school (45)	**24.5**	**4.1**	**40.4**	**2.7 (.27)**
**(Hetero) sexual debut**				
Yes (112)	**78.9**	**58.3**	39.1Φ	2.1(.17)
No (193)	**64.3**	**38.2**	31.3	2.1(.16)

*Bolded text indicates a statistically significant difference of p < .05.

Φ = significant at p = .08.

^‡^An unweighted abortion attitude scale was constructed as the sum of each participant’s responses to eight potential legal indications (see Table 4). Those who responded “under no circumstances” were scored as zero.

As expected, sexual debut increased with age, and awareness of peer pregnancy and peer abortion was similarly associated with age (See Table 3). Female gender (aOR 4.2,95% CI 2.4-7.2), (hetero)sexual debut (aOR1.76, 95% CI 1.1-3.0 ) and attending the prestigious magnet school (aOR 2.7 95% CI 1.4-6.3) were associated with reporting some type of abortion method in the multivariate model. Attitudes toward legal abortion were not associated with reporting an abortion method.

**Table 3:** Adjusted multivariate model of abortion method reporting by gender, sexual status, and school

	**Unadjusted Odds of Reporting an abortion method (95% C.I.)**	**Adjusted Odds of Reporting an abortion method (95% C.I.)**
Gender		
Girls (n = 214)	3.4 (2.1-5.5)	4.2 (2.4-7.2)
Boys (n = 131)	Ref	Ref
Sexual debut		
Yes	Ref	Ref
No	0.43 (0.3-0.62)	1.76 (1.1-3.0)
Knows a peer who has had an abortion		
Yes	2.2 (1.5-3.5)	1.62 (1.3-2.8)
No	Ref	Ref
School		
Technical middle school (n = 62)	0.35 (.16-.77)	Ref
Technical high school (n = 227)	0.88 (.47-1.6)	
Magnet high school (n = 45)	Ref	2.7(1.4-6.3)

A total of 271 responses were provided by 121 students, with girls contributing 78.5% of the reported methods described in this analysis. Among the entire sample, 13.5% listed one method, 12.5% listed two methods, and 8.0% listed three or more.

Herbal remedies were the most commonly cited method (44.0%). Among the forty references to herbal abortifacients, the most commonly specified (18.6%) was “marijuana tea” (*Cannabis sativa).* Roughly 14.0% of responses were non-specific “herbal remedies” (*remédios de ervas*), “herbal teas” (*chás de ervas*) or simply “herbs” (*ervas*). Homeopathic remedies were commonly cited: Buchinha do Norte (*Luffa operculata*), hallucinogenic mushroom tea (*Psilocibe semilanceata*), and cinnamon tea (*Cinnamomum zeylanicum*). Not as common were Carqueja (*Baccharis triptera*), Boldo (*Peamus boldus*), and sene (*Cassia angustifolia*) teas.

Of the thirty responses indicating ingestion of a medication, the most common pharmaceutical method cited was “Cytotec” (41.2%) the trade name of the prostaglandin analog misoprostol, a known abortifacient.

“There’s a method my friend talked about, that is taking ‘Cytotec’-- a stomach remedy--one pill oral and the other vaginal. The woman makes the uterus have contractions and abort.” –Female, Age 16, mixed race, Catholic/Afro Brazilian

Other pharmaceutical responses included erroneous references to ‘emergency contraception’ or the ‘day after pill.’ Eight of the 30 responses listing pharmaceuticals conflated emergency contraception^a^ with abortion methods, as below:

“I have seen my friends take a remedy called ‘the day after pill’ or something that causes a pregnant woman to menstruate and lose the child. My friends know this is not good for them, but due to lack of options and dialogue with their family they continue doing this”. –Female, age 16, mixed race, Evangelical

Responses also included various “cocktails” involving soft drinks, brands of non-prescription indigestion and cold remedies, alcohol, and tobacco. A large number involved Coca-Cola mixed with popular antacids, cold medicines, analgesics including “Sonrisal”™ or “Melhoral”™ as well as “Cebalena”™ with Cinnamon tea. Soft drinks were reportedly administered vaginally as well as orally:

“Throw Coca Cola in the vagina!!!” - Female, age 15, mixed race, Evangelical

The third category of abortion methods referenced foreign objects inserted into the uterus (7.0%). These included knitting needles, crochet hooks, wires, hangers, and vacuum cleaner hoses. Of these methods, crochet hooks were mentioned with the most frequency (5 of 12 responses).

“Puncture the placenta with a knitting needle”. – Female, age 16, white, Catholic

“Introducing a crochet hook. This method isn’t anything safe because it can cause perforations of the uterus”. – Male, age 16, white, Evangelical

Surgical procedures represented only 6.4% (n = 11) of the abortion methods given. These included references to antiquated techniques including caesarian section, or general references to “doing a surgery.” A number of younger students described techniques for termination of advanced pregnancies in violent terms including “crushing,” “stabbing,” “mashing,” “lethal injection,” “dismemberment,” and “dissection.” Some students referred to classical, but technically outdated, methods such as saline injection and sharp curettage.

“Introducing water with salt into the sac (in the belly of the woman) or with a probe. If not done correctly it can kill the woman”. – Female, age 14, mixed race, Catholic

Finally, the least frequently listed (3.5%) were those methods that referred to the use of physical force or attempting injury. For example,

“Punching yourself in the stomach.” – Male, age 14, white, no religion given

“Stick you [sic] finger in an electric socket. You run the risk of dying”. – Male, age 13, white, Kingdom of God

“Throw yourself down from a high place to interrupt a pregnancy. It is not safe”. – Female, age 13, no race given, Assembly of God

Only ten students (5.8%) referenced vacuum aspiration methods, the techniques for early abortion sanctioned by the Brazilian Ministry of Health and World Health Organization. Out of these, only two considered it a safe method. One girl, gave this nuanced response:

“Aspiration: a tube that sucks out the fetus, after the mother takes anaesthesia. It is safe if done in ideal conditions” (female, age 12, mixed race, Catholic)

### Perceived safety of abortion methods

Less than 60% of the abortion methods given included responses on the perceived safety of the method. Among those with safety information provided, 85.1% were deemed as unsafe and 7.0% as potentially unsafe. Only 7.9% of methods were assumed safe.

“Teas for making the menstruation come down are very effective, but they have many risks, such as haemorrhage”. – Male, age 14, white, Catholic

Students’ perceptions of abortion safety seemed to be largely informed by knowledge gained by word of mouth, and in some cases, peers’ personal experiences.

“I have a friend who drank marijuana tea to abort, and as a consequence she almost lost her uterus”. –Female, age 16, mixed race, Catholic

The safety issues reported for herbal remedies included – possible birth defects, death and infertility. Half of the adolescents (n = 7) who mentioned “Cytotec” either did not know about its safety or did not report their perceptions of its safety. Almost all of those who mentioned misoprostol safety regarded it as *always dangerous* or *sometimes dangerous*. One respondent wrote,

“Citotec [sic]- a pill that can be inserted into the vagina (it is an extremely risky method)”. –Female, age 16, Black, Catholic

“Citotec [sic] is not safe because it is a drug intended to treat ulcers”. – Female, age 16, mixed race, Catholic

Nine out of 11 references to surgical methods included comments indicating danger to a pregnant woman. Referring to sharp curettage, one student added

“There is a method of scraping the cervix of the uterus and the lining that supports the foetus. This method is dangerous, because the woman could become sterile or even die”. – Male, age 21, indigenous, Catholic

Of the eight students who mentioned aspiration, two perceived it as safe and six perceived it to be dangerous. A specific safety issue mentioned was a fear of retained products of conception.

“Aspiration, a piece of the foetus could be stuck inside the uterus”. – Female, age 15, white, Catholic

“Method by suction. It is not safe at all”. – Female, age 14, mixed race, Catholic

“It is a syringe that ‘sucks’ the foetus out of the uterus. It is very safe”. – Female, age 13, no race given, Catholic

### Awareness of clandestine abortion clinics

Two responses reflected a nuanced consideration of method as well as the physical and legal circumstances in their safety assessment:

“Suction…It is a little safe, if done in legalized clinics”. – Female, age 16, mixed-race, Catholic

“The safest method I’ve heard of is when it is done in a specialized clinic, but even then it is very painful”. – Female, age 13, white, Jewish

In addition to the responses that considered both technique and physical and legal safety issues, many implied a broad, but unspecified danger, such as:

“I think that no method of abortion is safe, because it could cause serious problems for the mother”. – Male, age 13, mixed race, Baptist

It was unclear whether the “serious problems” alluded to were health-related or referred to an assumption that pregnant women would be negatively affected by regret or legal complications, two ideas which were mentioned frequently.

### Legal abortion indications

Attitudes toward abortion indications among Rio de Janeiro students varied by circumstances of pregnancy. Legal termination in the case of rape was supported by a majority (55.6%) but ranged dramatically among schools (35.5% vs. 84.4%). Less than half of those queried (45.2%) supported legal abortion in the case of a life-threatening pregnancy. There were no significant attitudinal differences by gender, age, sexual debut, maternal education, exposure to sexual education, contact with health providers, internet use, or socioeconomic status. However school clusters were significantly different on six out of nine indications. High school students, and in particular the magnet school students, reported greater support for all indications (see Table 4) whereas 42.0% of the younger middle school students rejected all abortion indications.

**Table 4:** Percentages of students in favour of specific legal abortion indications by school (n = 335)

**When a woman or adolescent….**	**Total**	**Magnet high school (n = 45) %**	**Technical high school (n = 227) %**	**Technical middle school (n = 62) %**	**Significance level**
…has been raped	61.4 (212)	**84.4 (38)**	**64.3(146)**	**35.5 (22)**	**.000**
…has a pregnancy that threatens her life	50.1(173)	**66.7(30)**	**50.2(114)**	**37.1(23)**	**.003**
…has physical or mental health problems	34.2 (118)	31.1(14)	36.6(83)	29.0 (18)	.706
…has HIV/AIDS	30.1(104)	35.6(16)	30.8(70)	24.2(15)	.195
…has a foetus with physical or mental problems	24.3 (84)	**26.7(12)**	**29.5(67)**	**6.5 (4)**	**.006**
…is too poor to have a child	12.2 (42)	**17.8(8)**	**13.2(30)**	**4.8 (3)**	**.036**
…has four or more children	11.6 (40)	17.8(8)	11.5(26)	6.5(4)	.070
…does not want more children	8.4 (29)	**17.8(8)**	**7.0(16)**	**4.8(3)**	**.023**
…under no circumstances	20.6 (71)	**9.0(4)**	**17.0(39)**	**42.0(26)**	**.000**

*Bolded text indicates a statistically significance difference suggested by a p-value of less than .05.

## Discussion

Although only a third reported having had their own sexual debut (36.6%), adolescents in two schools were familiar with age peers who had had an unwanted pregnancies and abortions. Nevertheless, most students--and boys in particular--were reluctant to report or lacked knowledge of specific abortion methods. The low response (34.0%) to the method questions may reflect a genuine lack of information or prevailing social norms that preclude disclosure of “stigmatized knowledge” (i.e. information that is potentially discrediting to possess). Moore (2006) found that Brazilian adolescents often find it strategic to assert a type of public naïveté on sexual health matters to manage the stigma associated with possessing ‘too much’ knowledge[53]. Despite the relative lack of sexual, pregnancy, and abortion experiences in the peer networks of magnet school students, they were more inclined to admit contentious abortion method knowledge and abortion views. This may reflect a combination of an added willingness to disclose contrarian viewpoints, their (highly educated) parents’ beliefs, and/or greater social and cultural capital.

However, unlike previous research which found the use of herbal remedies to be defined euphemistically as menstrual regulators, the adolescents in this group readily identified their use as abortifacients. Some of the herbal remedies mentioned such as *Luffa operculata*, *Peamus boldo,* and *cannibus sativa* have demonstrated abortifacient properties in animals, but their effectiveness or safety in humans has not been established [54-56].

Findings from this exploratory study depart from the conclusions of earlier work that focused on hospitalized adolescents [27]. Our research does not confirm published findings of widespread knowledge and use of black market misoprostol among younger adolescents [31,32]. Rather, we find an awareness of a host of contraindicated and ineffective abortion methods. This suggests that there may be a large (heretofore unrecognized) population of youth who experiment with a broader array of ineffective methods, including both innocuous and harmful techniques. Students’ identification of other restricted substances (i.e. marijuana, hallucinogens, alcohol) as a means of pregnancy termination raises troubling questions about how legal restrictions on abortion may compound or foment other risk-taking behaviours.

Given students’ familiarity with a broad range of unsafe techniques, it is reassuring that most students accurately identify the health risks they entail. However adolescents’ misperceptions about certain techniques, such as aspiration and medication abortion, deemed safe by the World Health Organization and the Brazilian Ministry of Health, was unexpected [57,58]. The evidence-based legal abortion methods offered by the public health system are largely unfamiliar or poorly understood. Students’ assessment of the safety of abortion methods could be a result of misinformation or part of their broader critique of abortion as a practice.

As expected, adolescents tended to support existing legal restrictions. We found students’ attitudes toward legal indications varied by school, suggesting a potential role of local social norms and networks (and possibly testing effects) in shaping abortion attitudes. Perhaps the magnet school students may report more liberal attitudes toward abortion because the school is affiliated with the elite federal university, where more liberal views on abortion and sexuality are normative. Abortion attitudes were not shaped by contact with an older peer group, health providers, sexual education courses, internet exposure, or attendance at religious services (Additional file 1: Table S5). Elley (2011) have shown how social networks can influence sexual attitudes, mediating categories such as gender and class [59].

The extent to which student views on the law reflect actual beliefs or restatements of socially desirable positions is unknown. Adolescents may simply be aware of (and responsive to) the political or social climate in which these questions were posed. Although we hypothesized that the internet method would allow participants to be less influenced by social and environmental expectations, the clustering of attitudes by school, irrespective of age, gender, race and class suggests group norms were also at play [60]. The schools did not differ with regard to provision of sexual education.

As in all studies, this one has constraints which may impact its results. A sample of three urban schools precludes broad generalizability. The sample of schools was dictated by the structural barriers to establishing high-speed Internet connections in marginalized, low-income neighborhoods. At the time of this study, secondary school students comprised less than 40% of the total population aged 12–21 years of Rio de Janeiro. Therefore, many of Rio de Janeiro’s most vulnerable adolescents – such as those in the workforce, emancipated minors, or those who drop out due to pregnancy-- could not be included (Instituto Brasileiro de Geografia e Estatística 2001). In addition, parental refusal (29.1%) was a potential source of selection bias, as those adolescents exempted by parents may have differed from those included. However, because the study queries participants about the experiences of those of their social networks in addition to their own such selection bias is likely to be mitigated.

## Conclusions

This exploratory study offers insights into adolescent perceptions of a broad range of actual and imagined abortion methods. While it is difficult to assess to what extent knowledge and beliefs reflect actual behaviour, domain-specific knowledge is an important component of competent decision-making [61]. Given the lack of knowledge about safe and effective abortion methods reflected here, it is likely that abortion behaviour among adolescents may be more varied and involve more trial and error than prior hospital-based studies suggest. Adolescents’ experimental abortion behaviour may include innocuous combinations of over the counter medications, or illicit drugs, invasive techniques or deliberate self-harm. Despite so many adolescents knowing a peer who has aborted, few students report familiarity with the evidence-based abortion methods offered in the public health system. Moreover, some youth lacked a clear understanding of the distinctions between contraceptives, emergency contraception, and medication abortion methods.

Further research is needed to determine the origins of adolescents’ erroneous abortion information and the extent to which it influences their decisions and behaviour. Future studies should discern how students acquire information about archaic abortion techniques.

Prior research has suggested that, in practice, Brazilian adolescents terminate unwanted pregnancies despite legal restrictions limiting abortion access and irrespective of their stated beliefs about the legality or morality of abortion [62].

Worldwide, adolescents are more likely than adults to self-induce and suffer damaging sequelae from unsafe abortion procedures as a result of delayed help-seeking, financial barriers to safe services, and laws that preclude confidentiality for adolescents [4,33,63]

Our findings underscore the incomplete and inaccurate information with which Brazilian adolescents may make pregnancy and abortion decisions, and point to the need to further explore their channels of information and courses of action.

The Brazilian government has articulated a commitment to adolescents’ sexual and reproductive health through its sex education curricula, sexual violence prevention campaign, school-based condom distribution policy, and emergency contraception norms. Moreover it has begun treating unsafe abortion as a public health issue rooted in social inequality by drafting national technical guidelines for compassionate abortion care for adolescents [5].

Abortion knowledge has been found to correlate positively with abortion attitudes [64]. Providing knowledge to young people that is stigmatized in their social context is a sensitive matter. It requires on the one hand an age-appropriate, factual approach that facilitates development of social cognitive abilities, and emphasizes decision-making skills and includes methods for avoiding early, coerced or unprotected sex and pregnancy. Given the high rates of recurrence to unsafe abortion among Brazilian youth, pragmatic efforts must also be made to help adolescents accurately distinguish between *safe* and *unsafe* as well as *effective* and *ineffective* abortion methods. At the same time there is also an increasing call to move beyond a clinical and cognitive approach to sexuality and sexual health education towards an approach that acknowledges young people as sexual subjects and validates their experiences, emotions, and rights in relation to sexuality and abortion.

## Endnotes

^a^Notwithstanding any notoriety as an abortofacient, misoprostol is an important obstetrical drug with multiple uses including the treatment of postpartum hemorrhage, uterine atony, legal induced and incomplete abortion. It is registered for obstetrical use and sanctioned by the Ministry of Health in the 2005 and 2011 reproductive health norms.

^b^Emergency contraception consists of higher doses of hormonal contraceptives which can be taken up to 120 hours after sex to prevent pregnancy.

## Competing interests

LA and EMHM are former employees of Ipas, a global, non-profit women’s reproductive health and rights organization which previously manufactured and distributed manual vacuum aspiration instruments.

## Authors’ contributions

EMHM, CTH and LA designed the original study, crafted the measures, and designed the analyses. EMHM conceived of the article, conducted quantitative analyses and produced the first draft of the manuscript. AA and SH conducted literature review and qualitative data analyses. All authors contributed to the interpretation of these data and to the drafting of the manuscript. All authors read and approved the final manuscript.

## Pre-publication history

The pre-publication history for this paper can be accessed here:

http://www.biomedcentral.com/1472-6874/14/27/prepub

## Supplementary Material

Additional file 1: Table S5 Non-Predictors of Abortion Method Knowledge and Attitudes. Click here for file
