# Supplementary material for: Brazilian adolescents’ knowledge and beliefs about abortion methods: a school-based internet inquiry
Source: BMC Womens Health. 2014 Feb 13;14:27. doi: 10.1186/1472-6874-14-27 (PMC3924906; doi:10.1186/1472-6874-14-27)
Supplement: Additional file 1: Table S5 — Non-Predictors of Abortion Method Knowledge and Attitudes. [file 1472-6874-14-27-S1.docx]

**Table S5: Non-Predictors of Abortion Method Knowledge and Attitudes**

|  | *Have you heard or read about different methods to end an unwanted pregnancy?* | *When do you* *think termination of pregnancy should be legal* ?  Legal Abortion Attitude Scale  Mean (S.E.)  Range 0-8‡ |
| --- | --- | --- |
| **Total (% or mean)** | 34.0 | 2.1(.10) |
| **Religious Attendance** |  |  |
| Less than once a week | 29.4 | 2.2 (.15) |
| Once a week of more | 37.7Φ | 2.0 (.14) |
| **Most Recent medical Exam** |  |  |
| < 12 months ago | 39.1 | 2.2 (.13) |
| 1-2 years | 34.2 | 2.0 (.30) |
| > 2 years | 33.3 | 3.0 (1.7) |
| **Socioeconomic status** |  |  |
| low | 29.3 | 2.0 (.20) |
| medium | 35.4 | 2.1 (.15) |
| high | 36.7 | 2.1 (20) |
| **Attended Sexual Education Class** |  |  |
| No | 33.8 | 2.1 (.13) |
| Yes | 39.0 | 2.1 (.17) |
| **Age of Most Friends** |  |  |
| Younger or same age | 32.4 | 2.1 (.11) |
| Older | 40.0≈ | 2.0 (.22) |
| **How often connect to the internet** |  |  |
| 1-2 times | 30.0 | 1.7 (.26) |
| <1/month | 41.9 | 2.5 (.27) |
| 1-3/month | 43.2 | 2.0 (.30) |
| 1-6/week | 37.6 | 2.1 (.17) |
| daily | 37.2 | 2.5 (.36) |

Φ significant at .06 level, ≈significant at the .11 level
